# Unveiling cell-type-specific microRNA networks through alternative polyadenylation in glioblastoma

**DOI:** 10.1186/s12915-024-02104-8

**Published:** 2025-01-21

**Authors:** Mert Cihan, Greta Schmauck, Maximilian Sprang, Miguel A. Andrade-Navarro

**Affiliations:** Faculty of Biology, Johannes Gutenberg University Mainz, Mainz, Germany

**Keywords:** Glioblastoma, MicroRNA, Alternative polyadenylation, Stem cell, Single-cell RNA

## Abstract

**Background:**

Glioblastoma multiforme (GBM) is characterized by its cellular complexity, with a microenvironment consisting of diverse cell types, including oligodendrocyte precursor cells (OPCs) and neoplastic CD133 + radial glia-like cells. This study focuses on exploring the distinct cellular transitions in GBM, emphasizing the role of alternative polyadenylation (APA) in modulating microRNA-binding and post-transcriptional regulation.

**Results:**

Our research identified unique APA profiles that signify the transitional phases between neoplastic cells and OPCs, underscoring the importance of APA in cellular identity and transformation in GBM. A significant finding was the disconnection between differential APA events and gene expression alterations, indicating that APA operates as an independent regulatory mechanism. We also highlighted the specific genes in neoplastic cells and OPCs that lose microRNA-binding sites due to APA, which are crucial for maintaining stem cell characteristics and DNA repair, respectively. The constructed networks of microRNA-transcription factor-target genes provide insights into the cellular mechanisms influencing cancer cell survival and therapeutic resistance.

**Conclusions:**

This study elucidates the APA-driven regulatory framework within GBM, spotlighting its influence on cell state transitions and microRNA network dynamics. Our comprehensive analysis using single-cell RNA sequencing data to investigate the microRNA-binding sites altered by APA profiles offers a robust foundation for future research, presenting a novel approach to understanding and potentially targeting the complex molecular interplay in GBM.

**Supplementary Information:**

The online version contains supplementary material available at 10.1186/s12915-024-02104-8.

## Background

Glioblastoma multiforme (GBM) is recognized as the most aggressive and prevalent primary brain tumor in adults, posing significant challenges in neuro-oncology [1]. A key aspect in understanding the pathogenesis of GBM is identifying its cell of origin.

The observed heterogeneity in GBM points to various theories, including the prominence of glial progenitor cells and the stem cell hypothesis [2–4]. The latter suggests a subpopulation of stem cells within tumors that are characterized by their ability to propagate tumors, self-renew, differentiate into multiple lineages, express specific markers, exhibit low frequency, and resist drugs [5–8].

Recent research has revealed the presence of a subset of CD133 + radial glia-like cells in adult human glioblastomas, which exhibit characteristics similar to normal human fetal radial glia. These cells exist in various states, ranging from dormancy to active cycling, highlighting their significant role in the dynamics of tumor growth and maintenance [9].

Additionally, CD133 + cells, known for expressing genes associated with radial glial and neural crest cell development, have been implicated in the seeding of recurrent GBM tumors. These recurrent tumors display a diverse array of properties, including both neural and mesenchymal traits [10, 11].

The inherent capacity of radial glia-like cells to transdifferentiate demonstrates the plasticity and adaptability of tumor cells to microenvironmental cues, which complicates the dynamics of GBM progression and therapy resistance.

Experimental evidence has further elucidated the potential of these radial glia-like cells to differentiate into oligodendrocyte precursor cells (OPCs). Specifically, radial glia-like cells derived from human pluripotent stem cells have shown an enhanced capacity for oligodendrocyte lineage commitment, especially under the influence of specific growth factors, highlighting their adaptability and importance in neurogenesis [12]. Additionally, it has been found that pre-OPCs express neurogenic outer radial glia cell markers, indicating a lineage relationship and suggesting a complex interplay in the development of neural cell types [13, 14].

Within the dynamic landscape of the GBM tumor microenvironment, especially at the tumor borders, OPCs assume a critical role. These cells significantly contribute to the cellular architecture and intricacies of the tumor periphery, thereby influencing the neoplastic trajectory. OPCs, in concert with macrophages, construct a specialized microenvironmental niche that perpetuates the stemness of GBM cells and their resilience against therapeutic interventions [15, 16].

The cellular and molecular heterogeneity of GBM underscores the importance of gene regulation in its progression. Notably, microRNAs play a significant role in oligodendrocyte differentiation [17] and within the tumor microenvironment [18].

MicroRNAs like miR-219-5p, miR-219–2-3p, and miR-338-3p show heightened expression at the tumor fringes, indicating an abundant presence of oligodendrocyte lineage cells in these regions, a strong contrast to their sparse distribution within the central tumor mass [15, 19]. The role of these microRNAs is multifaceted and extends into various biological processes; they actively modulate the immune response, reshape the epigenetic environment, and alter the dynamics of GBM subpopulations. This broad spectrum of activity affects everything from cell proliferation to programmed cell death, significantly impacting tumor behavior and patient outcomes [20–23].

An additional critical aspect that must be considered to understand the mechanisms by which microRNAs regulate cellular transitions in GBM is the role of microRNA-binding through alternative polyadenylation (APA) modification of the 3' untranslated regions (UTRs) of target mRNAs. APA can alter microRNA-binding sites by shortening or extending the UTRs [24]. In cancer cells, the shortening of 3' UTRs compared to normal tissue may enhance mRNA stability and subsequently affect microRNA-mediated gene regulation [25].

However, a comprehensive understanding of regulatory mechanisms controlling microRNA binding cannot be explained by APA alone and needs to consider the interplay with other regulatory elements. This requirement for precision often leads to functional synergy with transcription factors, orchestrating a layered regulatory network [26, 27]. This complex interplay can be studied as the body of feed-forward loops (FFLs), sophisticated networks composed of microRNAs, transcription factors (TFs), and their target genes. FFLs play a crucial role in cellular proliferation, tumor formation, inhibition of cellular aging processes, and are used for classification of subtypes in oncological research [28–32].

In this study, we investigate the complex regulatory framework of GBM, emphasizing the intricate dynamics between microRNAs, APA, and FFLs. We seek to illuminate the role of microRNAs in influencing the fate of neoplastic radial-glia-like cells and their potential transdifferentiation to OPCs, employing single-cell RNA sequencing data. By examining APA dynamics and the shifts in microRNA-binding sites, we aim to understand how these molecular alterations impact cell fate decisions and transitions in GBM. We particularly focus on building cell-type-specific FFL networks, which will provide a detailed view of the co-regulatory interactions involving microRNAs and their contribution to the complex cellular ecosystem of GBM. Our analysis includes constructing pseudotime trajectories to model the progression paths of individual cell clusters within the tumor. This approach aims to unravel the subtleties of APA dynamics, cellular shifts, and the broader implications for cell fate and tumor heterogeneity in GBM.

## Results

### APA reveals an additional regulatory layer in GBM cellular heterogeneity

**Fig. 1:**
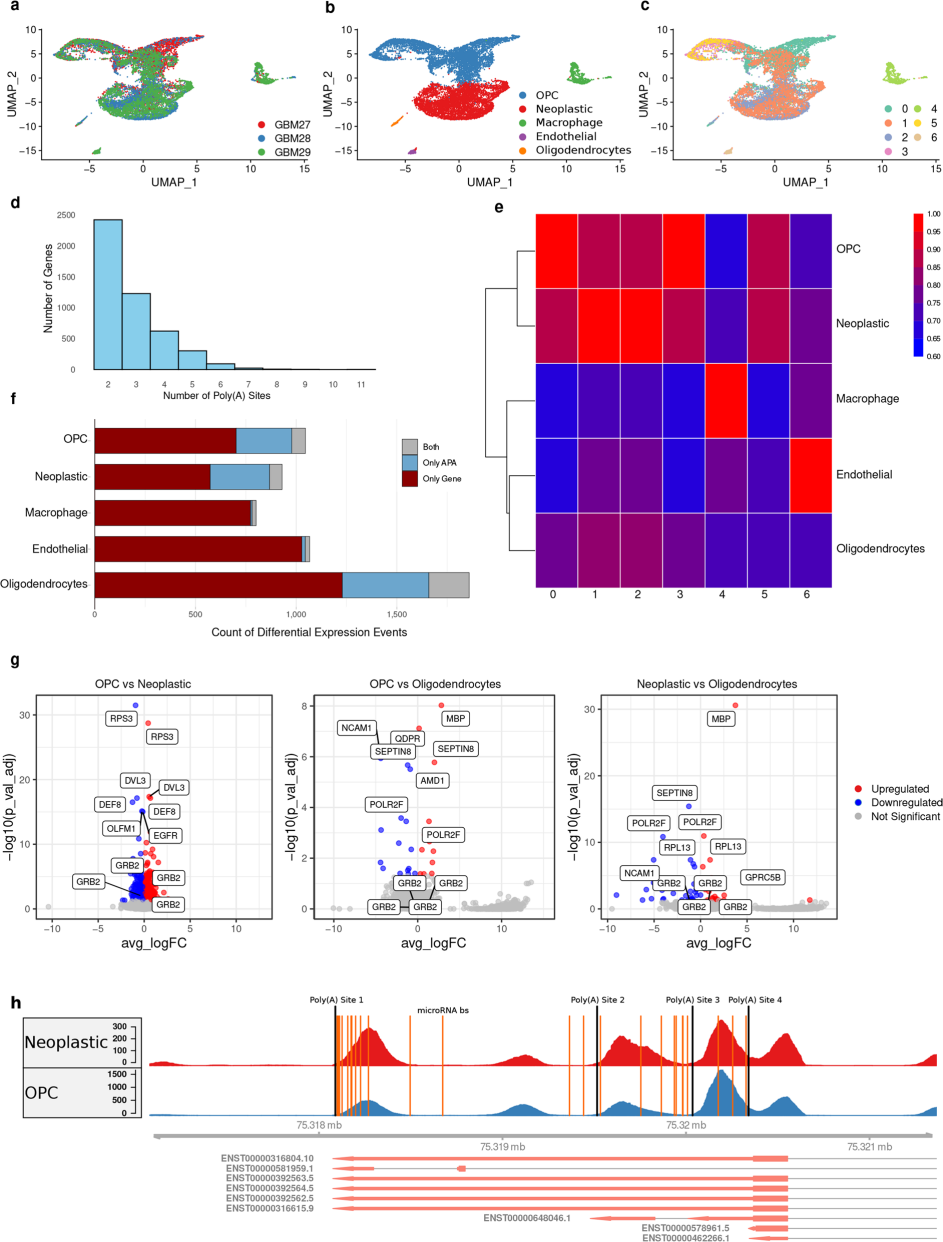
Single-cell clustering, APA events, and differential expression analysis. **a** UMAP plot showing single-cell clustering post batch effect correction, colored by sample origin. **b** UMAP plot depicting cell clustering results, colored by annotated cell types. **c** UMAP visualization of cell clusters and colored based on clustering results applied on APA matrix. **d** Histogram indicating the number of APA sites per gene. **e** Heatmap showing the overlap between APA and gene expression clusters. **f** Bar chart comparing the total differential expression event counts for each cell cluster vs. all other cell clusters. **g** Volcano plots showing differential gene expression differences between cell types. **h** Visualization of APA site variation for gene GBR2 across OPCs and neoplastic cells generated from BAM files

In order to study the role of APA in gene regulatory networks, including its dysregulation in a cancer setup, we chose to investigate single-cell RNA samples from GBM. Batch effect correction was critically applied to single-cell RNA sequencing data from three distinct GBM samples: GBM27, GBM28, and GBM29 (see “ Methods” for details). As delineated in Fig. 1a, this methodological step was imperative for normalizing across disparate samples, thereby ensuring a robust integration and comparative analysis of cell populations. Cell clustering based on gene expression profiles coupled with literature-derived cell type annotations resulted in the identification of distinct cellular contingents within the GBM milieu, specifically neoplastic cells, OPCs, endothelial cells, macrophages, and oligodendrocytes, as depicted in Fig. 1b (Additional file 1: Table S1). Notably, a substantial proportion of the cells were categorized as OPCs or neoplastic cells. To quantitatively assess the proximity of neoplastic cells to OPCs, we calculated the mean Euclidean distance from the neoplastic centroid to each cell in target clusters in PCA space. The results showed that the neoplastic-OPC distance (mean: 23.96, standard deviation [SD]: 7.12) was notably shorter than distances to other clusters: macrophages (mean: 50.15, SD: 10.9), endothelial cells (mean: 49.99, SD: 11.0), and oligodendrocytes (mean: 42.0, SD: 16.5). This marked reduction in distance highlights a distinct spatial alignment between neoplastic cells and OPCs, reinforcing a potential transitional relationship or shared lineage characteristics between these cell types.

In an innovative twist to traditional clustering approaches, cells were also clustered based on their APA profiles and colored in the gene expression-based UMAP. This analysis revealed an APA cluster 1, intriguingly interspersed between the OPC and neoplastic cells, as shown in Fig. 1c and further supported in Fig. 1e. This interposition suggests an overlap in APA patterns between these cells, despite their distinct placements when clustered by gene expression. While the majority of genes in the dataset exhibited a singular APA site, a total of 16.6% of genes featured multiple APA sites (Fig. 1d). To validate the APA events identified, we compared genes with multiple APA sites to PolyASite v2.0 [33] and PolyA_DB 3 [34]. Our results showed high overlap: 89.22% of genes with multiple APA sites in our dataset were also present in PolyASite, and 91.49% were found in PolyA_DB. Notably, only 142 genes were not captured by either database. These findings validate the robustness of our analysis and highlight the near-comprehensive coverage provided by these databases. This revelation hints at a layer of post-transcriptional regulation that warrants further investigation.

Upon examining the relationship between differential gene expression and APA site variability, it became evident that most genes that are differentially expressed are not subject to differential APA, and conversely, genes with differential APA are not predominantly differentially expressed (Fig. 1f). This apparent lack of overlap underlines a critical nuance; APA does not directly mirror gene expression changes but rather provides an additional layer of information, possibly affecting gene expression stability, localization, and protein translation efficiency. This realization emphasizes APA as a distinct, regulatory axis that complements traditional gene expression analysis.

Focusing on differential APA events among neoplastic, OPC, and oligodendrocyte clusters, it was observed that significant differential APA events do not necessarily correlate with large changes in gene expression in the clusters of OPC vs neoplastic, as exemplified in Fig. 1g (log2 fold change). This observation is pivotal, suggesting that while APA might not always dramatically shift gene expression levels, it could still be critically modulating gene function and cell state in a more subtle or context-dependent manner. Differential APA events between OPC and neoplastic cells were particularly observed for RPS3, DVL3, DEF8, EGFR, OLFM1, and GRB2.

The differential usage of the APA site 1 in GRB2, more frequently used in OPC than in neoplastic cells as evidenced in Fig. 1h, highlights APA’s potential impact on microRNA regulation. This trend might suggest a mechanism where shorter transcripts in OPCs resulting from preferential APA site usage reduce microRNA-binding opportunities, reflecting the interaction between APA and post-transcriptional regulation within GBM’s cellular diversity. To further explore the role of APA-regulatory genes in GBM cellular heterogeneity, we analyzed genes annotated under the Gene Ontology terms GO:0031124 (mRNA 3' processing) and GO:0110104 (mRNA alternative polyadenylation) to determine whether these genes exhibited significant differential APA events. Among the 29 identified APA-regulatory genes, 26 significant differential APA events were detected across 10 genes. These events were distributed across cell clusters, with 9 observed in the OPC cluster, 4 in the neoplastic cluster, 12 in the macrophage cluster, and 1 in the endothelial cluster (Additional file 2: Table S1-S6). Interestingly, only two of the APA regulatory genes, CPSF4 and ZC3H3, were among the highly variable genes used in the original PCA for clustering. However, none of these genes exhibited significant differential APA site usage, suggesting that their inclusion in the PCA was driven solely by expression variability rather than APA dynamics. This lack of representation among highly variable genes highlights a limited contribution of APA regulatory genes to the overall clustering process.

### Coordinated microRNA-binding site avoidance by APA highlights cell-specific regulatory strategies in GBM

**Fig. 2:**
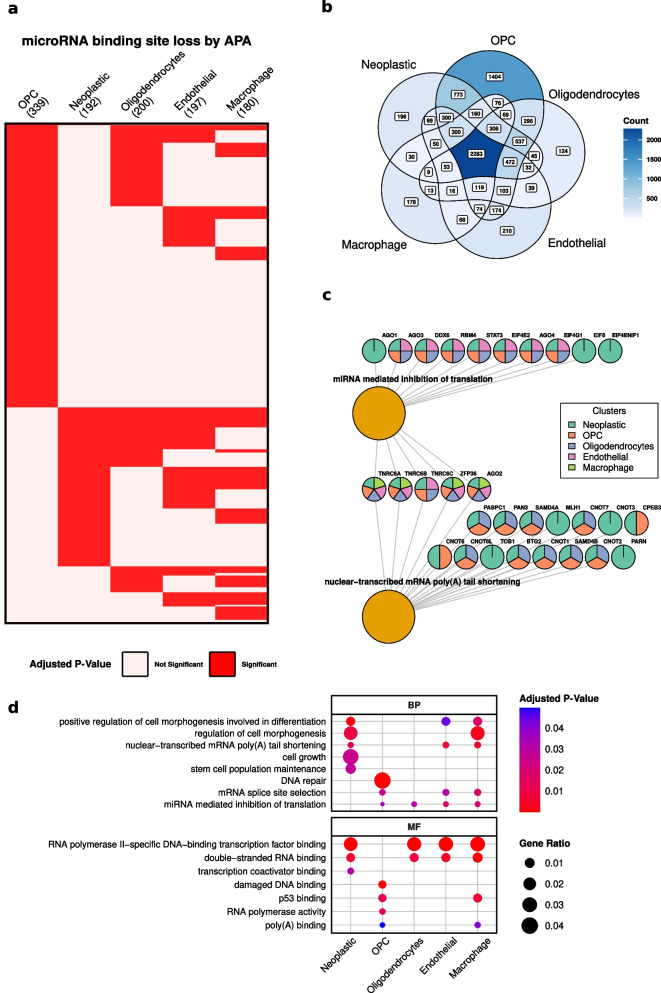
MicroRNA-binding site loss and GO term enrichment of APA events. **a** Heatmap displaying microRNA families with statistically significant loss of binding sites due to APA across cell clusters. **b** Venn diagram presenting the overlap of genes losing microRNA-binding sites in each cell cluster. **c** Network of GO terms and respective genes illustrated for different cell clusters. **d** Manually curated GO term enrichment results highlighting significantly enriched biological processes and molecular functions in different clusters

Investigation into the loss of microRNA-binding sites due to APA uncovers a sophisticated landscape of evasion strategies within the GBM tumor microenvironment. Analysis of cell-specific microRNA avoidance reveals that OPCs and neoplastic cells selectively evade distinct sets of microRNA families (Fig. 2a). For OPCs, out of the 912 microRNA families analyzed, 334 were avoided significantly by APA, whereas neoplastic cells show a significant avoidance in 192 microRNA families. This targeted avoidance is exclusive to each cell type, with no microRNA family found to be commonly avoided across both OPCs and neoplastic cells. However, both OPCs and neoplastic cells share significantly avoided microRNA families with other cell types (Fig. 2a). Such specificity in the regulatory landscape suggests that it may contribute to a complex adaptive mechanism of post-transcriptional regulation orchestrated by APA, which may underpin the cellular heterogeneity observed in GBM. This avoidance is not merely a binary event; several genes are involved in avoiding microRNA-binding sites across multiple clusters. Specifically, 773 genes are involved in avoiding microRNA-binding sites across both OPC and neoplastic clusters exclusively, suggesting a subset of regulatory processes that are critical to both cell types (Fig. 2b). In contrast, genes exclusively avoiding significant microRNA families in OPCs number 1404, whereas neoplastic cells have a unique set of 196 genes engaged in such avoidance.

The GO term analysis for these genes reveals a profound involvement in key processes and functions. In neoplastic cells, genes associated with “positive regulation of cell morphogenesis involved in differentiation” and “regulation of cell morphogenesis” present significant APA events. This suggests an APA-mediated emphasis on maintaining differentiation potential within neoplastic cells. Furthermore, “nuclear-transcribed mRNA poly(A) tail shortening” is a notable process, with 18 genes involved, which may indicate APA’s significant role in the regulation of mRNA stability and turnover, essential for the dynamic cellular states in neoplastic cells (Fig. 2c, Fig. 2d). Among the genes associated with this GO term, MLH1, CNOT3, TOB1, and PARN are exclusively associated with neoplastic cells.

Furthermore, in neoplastic cells, the significant enrichment of genes in processes such as “cell growth” and “stem cell population maintenance” points to a strategic use of APA in supporting the malignant phenotype (Fig. 2d). These genes are crucial in sustaining proliferative capacity and adaptability, with APA potentially acting as a regulatory buffer to mitigate the effects of oncogenic stress on these cells.

The molecular function analysis aligns with these biological processes. For neoplastic clusters, the significant evasion of microRNA regulation by APA is linked to “RNA polymerase activity,” “double-stranded RNA binding,” and “transcription coactivator binding.” These significant ratios indicate APA’s potential influence on transcriptional regulation, with possible implications for cellular identity and response to environmental cues. Moreover, functions like “damaged DNA binding” and “p53 binding” for OPC suggest a role for APA in modulating the DNA damage response and interactions with tumor suppressor networks (Fig. 2d).

### APA-mediated microRNA regulation in cell-type-specific networks and feed-forward loops

To further investigate the influence of microRNA regulation and APA on GBM, we delved into the intricacies of regulatory networks, focusing on simple feed-forward loops (SFFLs) and module feed-forward loops (MFFLs). SFFLs represent regulatory motifs that consist of three-node interactions among microRNA, TF, and gene, where the gene is regulated by both the TF and the microRNA, with additional interactions involving TF regulating microRNA, microRNA regulating TF, or both. MFFLs extend this concept by incorporating multiple such SFFLs into a larger, interconnected network, enabling a more complex and robust regulatory framework (see “ Methods” for details). This exploration is particularly pertinent given the observed enrichment of “transcription coactivator binding” in the GO terms, suggesting a complex interplay of transcriptional regulation within the tumor's cellular milieu.

In the explored GBM dataset, our analysis revealed a total of 3558 significant SFFLs. The neoplastic cluster accounted for the highest number with 382 SFFLs, followed by the OPC cluster with 277. Within these significant networks, 83 SFFLs in the OPC and 30 in the neoplastic cluster were associated with microRNA-binding site loss due to APA. The other cell types—oligodendrocytes, endothelial, and macrophages—presented 62, 37, and 198 SFFLs, respectively, reflecting their unique contributions to the complex regulatory milieu of GBM.

**Fig. 3:**
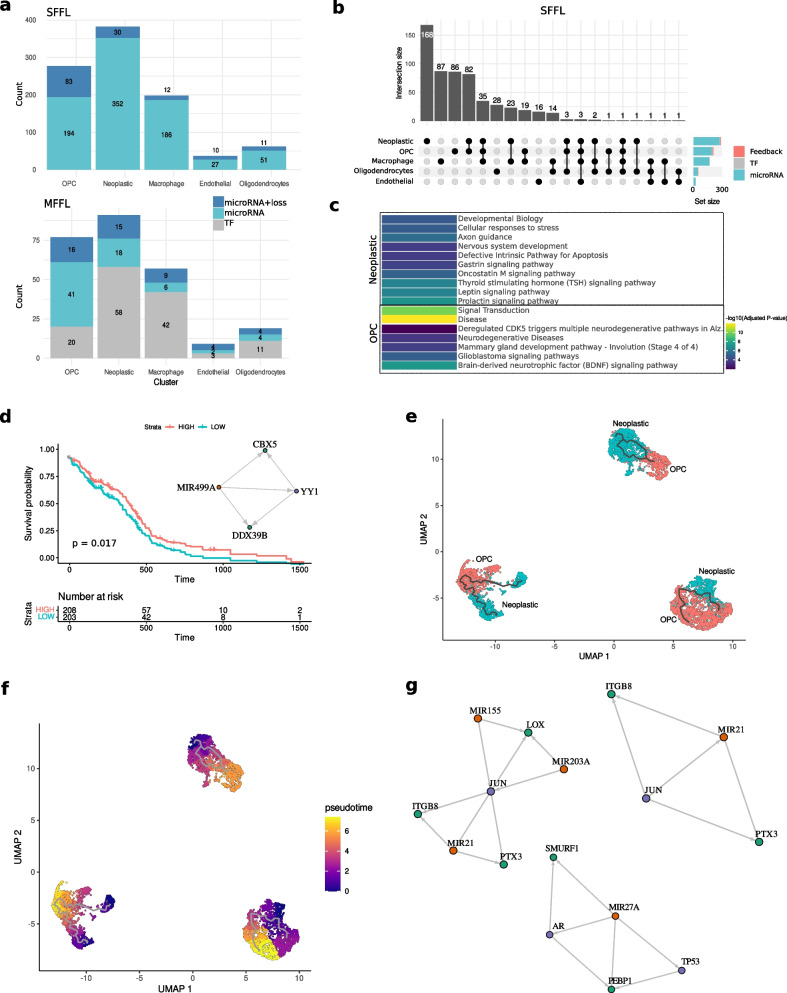
Network analysis and visualization of pseudotime trajectory. **a** Simple feed-forward loops (SFFLs) of microRNAs, transcription factors and target genes, as well as merged networks (MFFLs) for the same center node and their distribution among cell clusters. Number of microRNA families with significant loss of binding sites for respective cell cluster is indicated. **b** Upset plot showing the overlap of significant SFFLs among cell clusters. **c** Manually curated list of enriched pathways for genes and transcription factors involved in MFFLs in OPC and neoplastic clusters, computed with Reactome and Wiki pathways. **d** Kaplan–Meier plot based on GBM-TCGA data. **e** UMAP of filtered neoplastic cells and OPCs **f** Pseudotime trajectory analysis for neoplastic cell to OPC differentiation. **g** MFFL networks of microRNA, TF, and target gene containing genes involved in differential APA events and differential gene expression derived from pseudotime analysis and significant for neoplastic cells

Further dissecting the specificity of SFFLs within GBM, the upset plot analysis identified cell-type-specific loops: neoplastic cells exhibited 168 unique SFFLs, OPCs had 86, and macrophages showed 87. Additionally, an overlap was observed with 82 SFFLs shared between neoplastic and OPC cells, indicative of potential regulatory intersections between these cell types (Fig. 3a).

The analysis of MFFLs revealed 91 networks in the neoplastic cluster, the highest number across all cell types, and 77 in the OPC cluster. Neoplastic cells had a slightly lower count of central TF node MFFLs at 15, compared to 16 in the OPC cluster. In contrast, the OPC cluster showed a significant occurrence of microRNA-binding site loss due to APA in 41 microRNA-centric MFFLs. The counts for oligodendrocytes, endothelial, and macrophages were 19, 9, and 57 MFFLs, respectively, each with their own set of MFFLs affected by APA, illustrating the cell-specific regulatory strategies within the GBM tumor microenvironment (Fig. 3b).

Pathway profiling of OPC and neoplastic cell clusters, derived from MFFL analysis, reveals distinct biological pathways relevant to each cluster’s role in GBM. OPCs are associated with neurodegenerative diseases and glioblastoma signaling pathways, highlighting their versatile nature. The BDNF signaling pathway’s presence in OPCs may be pivotal for neuronal-like functions within the tumor milieu.

Neoplastic cells are involved in pathways like nervous system development and axon guidance, aligning with their invasive characteristics. The presence of the defective intrinsic pathway for apoptosis in neoplastic cells aligns with cancer’s typical evasion of programmed cell death, aiding in tumor survival (Fig. 3c).

To explore the potential prognostic relevance of MFFLs across different cell clusters, Kaplan–Meier survival analysis was conducted. Cox regression analysis was performed to account for potential confounding factors, including age, tumor immune dysfunction, and exclusion, with these variables included as covariates. This comprehensive analysis revealed seven MFFLs with *p*-values less than 0.05, underscoring their potential prognostic relevance in GBM. Among these, five belonged to the OPC cluster, one to the neoplastic cluster, and one to the macrophage cluster (Additional file 1: Table S4). Notably, the MFFL involving MIR499A, YY1, CBX5, and DDX39B in the OPC cluster not only demonstrated statistical significance with the lowest *p*-value of 0.017 (Fig. 3d) but also further distinguished by the loss of binding sites for the respective microRNA family due to APA in the OPC cluster exclusively, as previously identified in our cluster-specific analysis.

### Impact of APA and co-regulatory networks on neoplastic cell transdifferentiation to OPC in pseudotime analysis

To decipher the role of APA in the transdifferentiation of neoplastic cells to OPC and to understand the impact of co-regulatory networks, SFFLs and MFFLs, we embarked on a detailed modeling of the pseudotime trajectory. This trajectory encompasses cells positioned intermediately between neoplastic and OPC cell clusters, representing a dynamic spectrum of transdifferentiation.

The pseudotime trajectory analysis incorporated 5429 cells, with 2461 belonging to the neoplastic and 2968 to OPC cluster. UMAP analysis, based on gene expression profiles, delineated these into 3 distinct clusters consisting of 2051, 1716, and 1662 cells, respectively, each embodying a blend of neoplastic and OPC cells (Fig. 3e, Fig. 3f). This expression-based clustering was chosen to maintain consistency with the initial identification of neoplastic and OPC clusters and to track changes in gene expression patterns during the transitional phase. Root nodes, representing the origination points of differentiation to OPCs, were selected among the possible nodes provided by Monocle 3 as those most distant from OPC clusters (Fig. 3f).

A comprehensive differential expression analysis over pseudotime yielded 3974 significant differential expression events (*q*-value < 0.05, average log expression > 0.1). Among the genes identified as differentially expressed, 189 genes showcased significant differential APA events (adjusted *p*-value < 0.05, average log expression > 0.1) when juxtaposing OPC versus neoplastic cells. Differential APA events can alter regulatory elements, such as microRNA-binding sites, potentially driving or modulating the observed differential expression events. This underscores the intricate interplay between APA and gene expression, highlighting APA’s pivotal role in shaping regulatory landscapes during cell state transitions. These overlapping genes, characterized by differential alternative polyadenylation and expression, play crucial roles in significant SFFLs. Specifically, 23 SFFLs reported as significant in the neoplastic cluster and those reported for the OPC cluster are involved. Among these SFFLs, eight have been identified as subject to coordinated microRNA family regulation in the OPC cluster and four in the neoplastic cluster, underscoring the nuanced regulatory landscape (Table 1).

**Table 1:** Significant SFFLs comprising genes with differential expression in the pseudotime trajectory between neoplastic and OPC clusters and concurrent differential APA events

Cluster	MicroRNA family	Transcription factor	Target gene
Neoplastic	MIR153-1/MIR153-2	SP3	IRS2
Neoplastic	MIR155	JUN	LOX
Neoplastic	MIR155	STAT1	IL6ST
Neoplastic	MIR32	SP3	IRS2
OPC	MIR101-1/MIR101-2	CREB1	PEB1
OPC	MIR143	AR	PEB1
OPC	MIR214	TP53	PEB1
OPC	MIR27A	AR	PEB1
OPC	MIR27A	TP53	PEB1
OPC	MIR194-2	TP53	PEB1
OPC	MIR214	JUN	ITGB8
OPC	MIR214	JUN	PTX3

Additionally, these pivotal genes are components of two significant MFFLs in the OPC cluster (one TF-centric and one microRNA-centric) and seven significant MFFLs (four TF-centric and two microRNA-centric) in the neoplastic cluster (Table 2). This highlights the complex and integrated regulatory networks at play, driving the nuanced transitions and cellular dynamics inherent in GBM progression.

**Table 2:** Significant MFFLs comprising genes with differential expression in the pseudotime trajectory between neoplastic and OPC clusters and concurrent differential APA events

Cluster	MicroRNA(s)	Transcription factors/Target genes	Center node
Neoplastic	MIR155, MIR203A, MIR21	JUN, LOX, ITGB8, PTX3	TF
Neoplastic	MIR128-2, MIR129-1, MIR129-2, MIR140, MIR150, MIR155, MIR194-2, MIR223, MIR338, MIR494	STAT1, FAM172A, IL6ST	TF
Neoplastic	MIR101-1	BACH1, KLF6, PEBP1, TGFBR1	MicroRNA
Neoplastic	MIR101-2	BACH1, FOS, KLF6, PEBP1, WEE1, TGFBR1	MicroRNA
Neoplastic	MIR27A	AR, TP53,PEBP1	MicroRNA
Neoplastic	MIR143, MIR16-2, MIR27A, MIR21	AR, PEBP1, F2R	TF
Neoplastic	MIR214, MIR27A, MIR377	TP53, PEBP1, YWHAG	TF
OPC	MIR27A	AR, TP53,PEBP1, SMURF1	MicroRNA
OPC	MIR21	JUN,ITGB8, PTX3	TF

## Discussion

In exploring the landscape of GBM, our study delves into the diverse cellular environments of neoplastic CD133 + radial glia-like cells and OPCs. Central to this investigation is the role of APA in modulating the landscape of microRNA binding, studied at single-cell resolution. This mechanism unveils a hidden layer of post-transcriptional regulation, pivotal in contributing to the cellular heterogeneity characteristic of GBM. This approach has been instrumental in elucidating the complex interplay between APA and gene expression, highlighting APA's distinct influence over cellular behaviors and identities within the GBM matrix.

A significant finding from our study is the shared APA profile in cells transitioning from neoplastic to OPC states. The closer spatial alignment of neoplastic cells with OPCs in PCA space, as evidenced by significantly shorter Euclidean distances, aligns with existing hypotheses regarding a possible neoplastic-OPC transition. This observation suggests a regulatory mechanism, potentially orchestrated by APA, that prepares these cells for a shift in function or identity [35, 36]. The similarity in APA patterns, despite the differences in gene expression profiles, indicates a potential functional state or latent capacity that transcends the cells immediate phenotypic presentations.

The study further reveals a minimal overlap between differential APA events and gene expression changes, underscoring APA as an independent layer of regulatory information. We identified crucial genes that are differentially polyadenylated when contrasting neoplastic cells and OPCs, such as RPS3, DVL3, DEF8, EGFR, OLFM1, and GRB2, indicating their potential importance as biomarkers and for shifts in cellular identity. Specifically, the involvement of EGFR and GRB2 in the EGFR signaling pathway [37–39], the participation of DVL3 in the Wnt signaling pathway [40, 41], and the association of RPS3 with chemotherapy resistance [42] have been established, underscoring their potential roles in the GBM microenvironment concerning the differentiation of neoplastic cells into OPCs. The mechanisms regulating APA are multifaceted and involve interactions among cleavage and polyadenylation factors, splicing machinery, and transcription elongation processes. For instance, core components such as CPSF4 and ZC3H3 are known to influence 3' end processing efficiency and site selection, which may indirectly affect downstream gene expression and post-transcriptional regulation [36]. However, in our dataset, the lack of significant differential APA site usage in these genes indicates that their regulatory activity may not be cell-type-specific, at least in the context of glioblastoma heterogeneity. Furthermore, APA regulatory genes were notably absent from SFFLs/MFFLs in our analysis, suggesting that while these genes play crucial roles in global mRNA processing, they do not appear to participate directly in the co-regulatory networks shaping cell identity in glioblastoma.

The identification of specific microRNA families selectively evaded by different cell types emphasizes the need for precise control over microRNA interactions. In neoplastic cells, APA-regulated genes are predominantly associated with cell growth and stemness, signifying APA’s role in driving the proliferative and adaptive nature of these tumor cells. In OPCs, APA produces mRNA variants of DNA repair genes that evade microRNA repression, potentially strengthening their genomic stability capabilities. This enhanced ability to repair damage could confer a higher level of resistance to chemotherapeutic agents, contributing to the tumor’s resilience [43, 44]. Therefore, APA may represent a critical factor in maintaining the integrity and robustness of the tumor, particularly considering OPCs roles in the tumor microenvironment and chemo-resistance [15, 16].

The overlap in APA patterns observed in the UMAP clustering (Fig. 1C) reflects global transcriptomic similarities in polyadenylation usage, particularly during the transitional phase between neoplastic cells and OPCs. This clustering emphasizes shared regulatory mechanisms and gene expression profiles that may contribute to a common functional state. In contrast, the microRNA avoidance analysis (Fig. 2A) focuses on specific APA events that alter microRNA-binding site availability, shedding light on cell-type-specific regulatory consequences. While the shared APA patterns observed in UMAP reflect a broader regulatory foundation, the microRNA avoidance analysis highlights how certain APA events drive fine-tuned adaptations in each cell type’s regulatory landscape. This distinction underscores the complementary nature of these analyses in understanding both the global and specific impacts of APA.

The critical impact of alternative polyadenylation in altering microRNA interactions and contributing to increased glioma cell migration by evading the binding sites of the miR-124 family has been previously demonstrated [45]. In our research, this family is notably identified for its substantial loss of binding sites in OPCs and is instrumental in the formation of MFFLs. In the broader landscape of glioblastoma research, our findings resonate with studies highlighting the complex interplay of APA, microRNA, and their impacts on cellular dynamics [46–48].

To delve deeper into the mechanistic aspects of microRNA regulation, we conducted a comprehensive network analysis which allowed us to further investigate the complex relationship with TFs, target genes, and APA. The computation of FFLs as a basis for exploring the interaction of microRNAs and transcription factors with target genes to fine-tune gene regulation has been a widely studied topic in the literature. Numerous studies [31, 49–51] have shown the importance of these regulatory loops in understanding how transcription factors and microRNAs interact to regulate gene expression. The integration of SFFLs into MFFLs within our study has elucidated a more comprehensive understanding of the regulatory dynamics in GBM. This approach has revealed the prominence of cell-type specificity in these networks, notably in the context of genes associated with neoplastic cells, which are found to be enriched in critical pathways such as the defective intrinsic pathway for apoptosis. This observation not only highlights the role of these networks in sustaining the malignant phenotype of GBM cells but also their contribution to resistance against apoptotic mechanisms [52].

In contrast, the genes implicated in OPC-specific MFFLs exhibit a distinct association with GBM signaling pathways. This divergence underscores the unique functional roles and identities of different cell types within the tumor microenvironment, reflecting the complex cellular architecture and the multifaceted nature of GBM.

Further scrutiny of our data reveals a significant role for key microRNAs, including miR-21, renowned for its oncogenic potential and involvement in chemoresistance [53–56]. The emergence of miR-21 as a central figure in OPC-specific MFFLs not only validates our methodological framework but also expands the understanding of its role within the GBM microenvironment. Additionally, the presence of miR-138–2, associated with high expression levels in oligodendrocyte differentiation [12], in significant OPC MFFLs, further substantiates the importance of our network analysis. The observed significant loss of binding sites for these microRNAs in OPCs, driven by APA, underscores a complex regulatory adjustment, highlighting the dynamic interplay between APA and microRNA regulation.

The utility of MFFL computation is further underscored not only by the significant loss of binding sites for microRNA families within neoplastic cells, attributable to APA, but also by their potential for utilization in survival analysis, enhancing our understanding of prognostic indicators in GBM and providing a foundation for hypothesis generation that warrants further investigation. Our utilization of pseudotime trajectory analysis has been pivotal in revealing that genes differentially expressed during the transition from neoplastic cells to OPCs are actively involved in MFFLs. MicroRNAs such as miR-21 reemerge as crucial elements in these networks, underscoring their influential role in GBM pathology across various regulatory scenarios. Given that the roles of transcription factors can vary depending on the cellular context, our resource of cell-type-specific SFFLs and MFFLs provides a flexible framework for exploring these interactions in a context-dependent manner, without assuming a universally fixed role for transcription factors as activators. Instead, the direction and impact of these interactions are determined by the specific expression patterns and regulatory relationships in each cell type and context. Our MFFL computation has elucidated key aspects of regulatory networks, considering the role of microRNA networks in oncogenesis and tumor suppression [32, 57], pan-cancer relevance [58], potential as drug targets [29, 58, 59], and their demonstrated ability to uncover glioblastoma heterogeneity and cell state transitions.

One limitation in studying SFFLs and MFFLs is the reliance on interaction databases, which can be error-prone or incomplete. To mitigate this, we focused on experimentally validated interactions and conserved interactions across species. However, the networks may still be incomplete, and further refinement may be necessary to enhance their accuracy and comprehensiveness. Additionally, the absence of comprehensive microRNA expression data, particularly in single-cell RNA sequencing, may limit the interpretation of certain interactions.

Our study’s primary focus on neoplastic CD133 + cells and OPCs may limit the broader understanding of GBM's cellular complexity. Additionally, the static nature of our research overlooks the dynamic changes that GBM undergoes over time, emphasizing the need for longitudinal studies, for example, APA profile changes in response to therapy. While we emphasized APA and microRNA regulation, other post-transcriptional mechanisms remain unexplored. Experimental validation and the incorporation of emerging single-cell sequencing technologies are necessary to strengthen the biological relevance of our findings.

In summary, our research provides an in-depth analysis of the cellular dynamics within GBM, focusing on the interplay between cell-type-specific APA, microRNAs, TFs, and target genes. We have successfully constructed and analyzed networks that capture the complex regulatory interactions within GBM, particularly highlighting the unique APA profiles of OPCs and neoplastic CD133 + radial glia-like cells. Our study unravels the subtle yet impactful ways in which APA contributes to cell identity and transformation in GBM, emphasizing the role of microRNA and TF in these processes. These insights provide a comprehensive view of the molecular intricacies that drive the heterogeneity and progression of GBM, offering a valuable resource for future research and potential clinical applications.

## Conclusions

In conclusion, our study has effectively mapped the APA landscape in GBM, highlighting its role in the transition between neoplastic cells and OPCs. Utilizing single-cell RNA sequencing data, we identified APA profiles crucial for this cellular transition, providing insights into the post-transcriptional regulation involving microRNAs, transcription factors, and gene networks.

Our workflow pinpointed genes undergoing significant APA changes and subsequent microRNA-binding site loss linked to stem cell maintenance and DNA repair, essential for understanding GBM’s adaptability and resilience. The elucidation of APA’s influence on microRNA-transcription factor-gene networks has revealed new dimensions of GBM’s molecular complexity, revealing both potential therapeutic targets and marker genes for survival analysis.

This research not only sheds light on the regulatory dynamics within GBM but also sets the stage for future studies aiming to exploit APA mechanisms for therapeutic innovation, aligning with the goals of precision medicine in oncology.

## Methods

### Dataset and single-cell clustering

We procured single-cell RNA sequencing data from three glioblastoma samples (GBM27, GBM28, and GBM29), obtained from GSE139448 [60]. The single-cell datasets were processed using CellRanger [61], employing the “mkfastq” function for generating FASTQ files, the “count” function for aligning reads and quantifying gene expression, and the “aggr” function for merging data across samples. Initial quality control steps involved filtering cells based on mitochondrial reads (< 10%) and selecting for RNA counts in the range of 2000 to 10,000. This resulted in 13,600 cells eligible for further analysis using Seurat v3.2.3 package [62]. Normalization and variable feature identification were conducted using Seurat package. Principal component analysis was utilized for dimensionality reduction, with the number of significant components determined via elbow plot methodology, resulting in 17 principal components. We computed Euclidean distances from the neoplastic cell centroid to each cell in other clusters, thus quantifying their spatial proximity in PCA space. To correct for batch effects across samples, we employed the “FindIntegrationAnchors” function, to integrate data from the three distinct samples. Unsupervised clustering was then executed using “FindNeighbors” and “FindClusters” functions. Cell-type annotations were assigned by both genetic expression profiles and known markers (Additional file 1: Table S1). Differential gene expression was computed using the “FindAllMarkers” function, enabling the identification of unique molecular signatures across different cell clusters.

### MicroRNA interactions

In assembling our high-quality microRNA-target gene interaction dataset, we included reported interactions from TRANSFAC [63], enriched with experimentally supported interactions from DIANA-TarBase v8 [64] and miRTarBase [65]. We then cross-referenced these with predictive binding sites from TargetScanHuman 8.0 (TSH) [66], supplemented by predictions from broadly conserved microRNAs and their binding sites in TSH. This resulted in a dataset comprising 2,908,279 predicted binding sites for 912 microRNAs, targeting 12,612 genes. To enhance compatibility with current genomic studies, we used UCSC’s liftOver [67] to update these binding sites from hg19 to hg38. This comprehensive dataset stands as a critical resource for studying microRNA-mediated gene regulation.

### Alternative polyadenylation events

In our study, the APA matrix was computed using the SCAPE program with its default parameters [68]. The APA matrix represents expression values quantified for each polyadenylation site within a gene, providing a finer resolution of polyadenylation usage across samples rather than summarizing expression at the gene level. Additionally, the “FindDE” function was employed to identify differential APA events. This function applies DEXSeq-based analysis, comparing APA usage for each cell cluster against all other clusters to identify cell type-specific APA patterns. Statistical significance was determined using adjusted *p*-values < 0.05. APA events meeting this threshold were considered statistically significant and prioritized for downstream analyses, ensuring a rigorous and reliable identification of cell type-specific APA usage (Additional file 2: Table S1-S5). We focused our analysis on genes expressed in at least 10% of the cells in each cluster. This threshold ensured that our analysis was based on genes with a significant presence in each cellular subset, providing a more accurate reflection of the APA dynamics within the different cell clusters.

### MicroRNA avoidance analysis

We quantified the avoidance of microRNA regulation through APA for each cell cluster by using the computed APA expression matrix and annotated microRNA-binding sites. The analysis involved calculating the ratio of total microRNA-binding sites lost versus retained for each microRNA family within each cell cluster. To assess statistical significance, we executed 10,000 permutations, where the cell assignments were randomly shuffled across all clusters while maintaining the original cell counts. In each permutation, the ratio was recalculated, and the incidence of ratios higher than the observed ratio was tracked. The resulting *p*-value, derived from the frequency of higher ratios divided by the total permutations, indicates the significance of microRNA regulation avoidance in each cell cluster. To account for multiple testing, *p*-values were adjusted using the Benjamini–Hochberg [69] method, with a significance threshold set at 0.05 (Additional file 1: Table S2).

### Construction of feed-forward loops

To construct the FFL networks, our methodology integrated four types of directed regulatory interactions: microRNA to TF, microRNA to gene, TF to gene, and TF to microRNA. The microRNA to TF and microRNA to gene interactions were derived from our compiled microRNA-target gene interaction dataset. For TF to gene and TF to microRNA interactions, we utilized TRANSFAC [63] and TransmiR v2.0 databases [70].

We defined a list of transcription factors as defined in [71] and extracted validated and conserved microRNA to gene/TF interactions. We merged these with TF to microRNA interactions from TransmiR [70] and TF to gene interactions from TRANSFAC [63].

From this background graph, we extracted all possible three-node interactions between microRNA, TF, and gene, categorizing them as simple feed-forward loops (SFFLs). We differentiated these SFFLs based on their central nodes: microRNA-centric (microRNA to gene and microRNA to TF, followed by TF to gene), TF-centric (microRNA to gene and TF to microRNA, followed by TF to gene), and feedback loops (microRNA to gene, TF to gene, microRNA to TF, and TF to microRNA). Subsequently, we identified module feed-forward loops (MFFLs) by merging all significant SFFLs that originated from the same central node such as a specific microRNA. Further, we set the constraint that nodes of these SFFLs must be expressed in at least 10% of the cells belonging to the cluster. This approach allowed us to discern broader regulatory patterns and understand the influence of individual entities like a specific microRNA on multiple pathways in GBM. While missing small RNA expression data, especially for microRNAs, is a known limitation in single-cell RNA sequencing, our use of MFFLs helps mitigate this challenge by integrating multiple SFFLs into broader networks. This approach enables the capture of biologically meaningful interactions even when individual microRNA data are incomplete, allowing us to interpret regulatory dynamics driven by APA and transcription factors with greater robustness.

### Significance testing of SFFLs and MFFLs

To ascertain the significance of SFFLs in diverse cell types, we scored networks comprising microRNA, TF, and gene nodes. In order to score computed FFLs, we applied the methodology prior demonstrated by [58]. Node scores, based on adjusted *p*-values from differential expression analysis, were computed by comparing the expression of a gene in a specific cell cluster versus in all other cells. These scores underwent inverse normal cumulative distribution transformation. Edge scores were computed using Fisher transformation of Pearson’s correlation z-scores between nodes. The average of node and edge scores constituted the overall SFFL score. Due to limitations in capturing small RNAs in single-cell RNA data, if microRNA scores were absent, we calculated the score using the remaining nodes and edges. However, to address the sparsity of microRNA expression data, we enhanced the gene expression matrix by employing the PPMS software [72], which profiles primary microRNAs with cell-type specificity, concentrating on the inclusion of those microRNAs that were absent in the initial gene expression dataset.

To assess the significance of the SFFLs, we permuted the network 10,000 times by selecting three random nodes—one microRNA, one TF, and one target gene—from the same cell cluster as the original SFFL. For each permutation, we computed the overall SFFL score again by calculating the node scores and edge scores for the randomly selected nodes. The randomized overall scores were then compared to the original SFFL score. The *p*-value for each SFFL was determined by calculating the ratio of random scores greater than or equal to the observed score. Finally, the Benjamini–Hochberg FDR correction [69] was applied to account for multiple testing, with a significance threshold of 0.05 (Additional file 1: Table S3). All significant SFFLs within a specific cluster were merged together based on the center node, which makes up the MFFLs for that cluster, providing a consolidated view of the regulatory networks for that cell type (Additional file 1: Table S4).

### GO term enrichment for genes avoiding microRNA regulation by APA

For the Gene Ontology (GO) term analysis, we conducted an examination specifically on genes identified as avoiding microRNA regulation by significant microRNA families. This analysis was restricted to genes pertinent to each individual cell cluster. Utilizing the “enrichGO” functions from ClusterProfiler [73], we focused on biological processes (BP) (Additional file 1: Table S6) and molecular functions (MF) (Additional file 1: Table S7) to discern the biological and molecular underpinnings influenced by this evasion. This approach allowed us to gain insights into the unique biological processes and molecular functions affected by the avoidance of microRNA regulation in each specific cell cluster context.

### Pseudotime trajectory

Utilizing Monocle 3 [74], we computed pseudotime trajectories, specifically targeting cells from the neoplastic cell to OPC clusters to model a potential transdifferentiation (Additional file 1: Table S5). To analyze differential expression over time, we conducted graph-autocorrelation analysis using the “graph_test” function. This was preceded by the application of the “estimate_size_factors” function, adhering to its default settings.

### Kaplan–Meier survival analysis

Kaplan–Meier survival analysis was performed using TCGA-GBM clinical and gene expression data, processed with TCGAbiolinks [75], survival [76], and survminer packages in R. Clinical data pertinent to survival was extracted, and gene expression data were normalized using DESeq2’s [77] variance stabilizing transformation. Cox regression analysis was conducted using the “coxph” function [76], incorporating gene expression values, age at diagnosis and TIDE-derived exclusion and dysfunction scores [78] as covariates, as they reflect the functional state of immune cells and the extent of immune evasion in the tumor microenvironment.

The coefficients for the respective genes obtained from the Cox regression model were combined with their expression values to stratify patients into high-risk and low-risk groups. Kaplan–Meier survival curves were then generated for these stratified groups, and statistical significance was assessed with a threshold of *p* < 0.05 (Additional file 1: Table S4). This approach, as demonstrated by, e.g., [79, 80], allowed us to explore the prognostic significance of MFFLs in the context of multiple clinical and molecular factors.

## Supplementary Information

Additional file 1: Table S1. Marker genes used for cell annotation. Table S2. Adjusted *p*-values for microRNA biding site loss for each microRNA family. Table S3. SFFL networks. Table S4. MFFL networks. Table S5. Differential expression results for pseudotime trajectory from neoplastic cells to OPCs. Table S6. GO enrichment results (BP). Table S7. GO enrichment results (MF).

Additional file 2: Differential alternative polyadenylation results computed with SCAPE for the cluster of neoplastic cells (Table S1), OPCs (Table S2), oligodendrocytes (Table S3), macrophages (Table S4) and endothelial cells (Table S5). Table S6. Differential APA events for genes regulating 3’ end processing of mRNAs.

## Data Availability

All data generated or analyzed during this study are included in this published article, its supplementary information files, and publicly available repositories. The single-cell RNA sequencing data analyzed in this study is publicly available at GEO under the accession number GSE139448 [60], as referenced in the “ Methods” section.

## Abbreviations

GBM: Glioblastoma multiforme
OPCs: Oligodendrocyte precursor cells
APA: Alternative polyadenylation
UTRs: 3' Untranslated regions
FFLs: Feed-forward loops
TFs: Transcription factors
SFFLs: Simple feed-forward loops
MFFLs: Module feed-forward loops
TSH: TargetScanHuman 8.0
GO: Gene Ontology
BP: Biological processes
MF: Molecular functions
